# Editorial: Understanding the Role of Non-verbal Displays in Politics

**DOI:** 10.3389/fpsyg.2022.892103

**Published:** 2022-04-26

**Authors:** Carl Senior, Patrick Alan Stewart, Delia Dumitrescu

**Affiliations:** ^1^Aston University, Birmingham, United Kingdom; ^2^University of Arkansas, Fayetteville, NC, United States; ^3^Heidelberg University, Heidelberg, Germany

**Keywords:** politics, non-verbal behavior, social science research, fake news, populism and democracy, facial displays, signals [Behav Ecol]

## Introduction

Approaches to understanding political figures focusing solely on their words fall short. Even the most verbally articulate of spokespersons with the most well-developed of scripts fail to convince audiences when their presentation is awkward or not credible. At the same time, less verbally articulate individuals have at times seized the imagination, and followership, of those enraptured by their charismatic speechmaking, despite an obvious lack of knowledge. With some highly scripted leaders, it may become apparent that there is sometimes an incongruence between the verbal message conveyed and the non-verbal signaling accompanying it (Bucy and Newhagen, 1999). More so than other public figures, politicians whose behavior deviates slightly from the norm may be scrutinized even more closely and deemed significantly inappropriate by those not belonging to their political in-group (Stewart and Hall, 2016).

In short, when it comes to leadership and persuasion our politicians need to be aware of what they say, how they say it and to whom they direct their message. Yet despite the importance of understanding the totality of political messages there remains a surprising lack of research into the role played by non-verbal channels in conveying political messages. Such paucity is surprising when considering the importance of accurately decoding, as well as the potential aftermath of misunderstanding political messages (e.g., Lazer et al., 2018).

The lack of work exploring the non-verbal domain is perhaps surprising as, when individual political figures are scrutinized, it becomes apparent that each exhibit idiosyncratic and potentially predictable non-verbal behavioral repertoires (Patterson, 2017; D'Errico, 2019). It is therefore possible to examine and identify deviations in their portfolio of non-verbal behaviors and infer causes for such deviations (Bucy and Stewart, 2018). Considering how, when, and why such behavioral deviations occur in response to questions during interviews and speeches provides an opportunity to examine honest signals of behavioral intent that may, or may not, cohere with the spoken narrative (Choi et al., 2016; D'Errico et al., 2022; Bull, 2023). Thus, focusing on non-verbal displays by politicians provides us with a natural laboratory to examine communicative behaviors when placed in their most natural social setting. However, the study if non-verbal cues and signals from the body and face may provide salient and reliable information evaluating political leaders but this information is affected by a multitude of other factors and is not a straightforward affair (Poggi et al., 2013).

In this Research Topic, Murray and Carroll found preference for leaders possessing capacity cues indicating physical formidability, even after controlling for gender stereotyping, political ideology, and a host of socio-demographic control variables. They found this as related to greater preference for male over female leadership in time of intergroup threat. Likewise, Watkins et al. in their pre-registered experiment found that partisanship and type of media—whether partisan or non-partisan—affected the influence of facial cues of dominance and competence on judgments regarding leadership.

The influence of nonverbal signals by leaders can likewise be seen as affected by, and affecting, the political context. Stewart and Svetieva found that in the 3 weeks prior to the 2016 United States presidential election a one-minute video experimentally presenting Donald Trump's micro-expressions of fear had a generally positive effect on perceptions of this competence and trustworthiness in comparison with its absence, but in the days just prior to the election participants were largely unaffected by this form of contemporaneous information. For their part, Keating et al. found that, using short (30 s) silent videos of speeches, that perceptions of formidability (competence and power) and receptivity (warmth and attractiveness) were independently associated with judged charisma, although the effect of trustworthiness and authenticity were either weak or negligible. Furthermore, the resultant perceived charisma provides for influence over controversial group decisions—which is the very essence of politics at any level.

Given the massive volume of visual communication circulating on the internet, and especially on social media, understanding how non-verbal displays are used to persuade is of utmost importance. As part of this Research Topic Senior et al. report a case study on Boris Johnson's media performance in the run up to the 2019 UK general election—an event which resulted in a seismic shift in the UK political landscape. Valmori et al. investigate gendered face-ism in the presentation of Finish and Italian politicians. Their results point to a potential divide between the way women politicians present themselves on their public Facebook accounts as compared to their private ones. In the private Facebook sphere, women are more likely than men to display facial prominence, and therefore signal agentic qualities; there are, however, limited differences when it comes to public profiles. Compared to previous research, this study suggests one, that gendered differences in facial displays may be waning when looking at public social media profiles, and two, that women politicians may feel pressured to display different images on their public as compared to their private accounts.

Dumitrescu and Trpkovic's analysis of the Covid-19 disinformation messages indexed by the International Fact-Checking Network (IFCN) in Europe in April and May 2020 shows that non-verbal displays are utilized to enhance the negative emotional punch of the posts. Such messages appear to have at least two strategies: one, to ramp up viewers' negative reactions by portraying those in power smiling and with open body poses in the face of the tragedy of the pandemic; and two, by utilizing negative emotional displays in association with laying the blame on governments and private companies. Non-verbal displays are also used by disinformation producers to mark clear the divide between ordinary people (portrayed sporting closed, contractive body poses and negative facial expression), and those with political of financial power (shown to display open, expansive body poses). Taken together the papers within this Research Topic provide a comprehensive insight into how politicians use non-verbal displays to convince the voting masses.

## Author Contributions

All authors listed have made a substantial, direct, and intellectual contribution to the work and approved it for publication.

## Conflict of Interest

The authors declare that the research was conducted in the absence of any commercial or financial relationships that could be construed as a potential conflict of interest.

## Publisher's Note

All claims expressed in this article are solely those of the authors and do not necessarily represent those of their affiliated organizations, or those of the publisher, the editors and the reviewers. Any product that may be evaluated in this article, or claim that may be made by its manufacturer, is not guaranteed or endorsed by the publisher.
